# Impact of dental caries on the quality of life of adolescents: A systematic review and meta-analysis

**DOI:** 10.1590/1980-549720250018

**Published:** 2025-05-02

**Authors:** Carlos Roberto Botelho-Filho, Giuliana Martina Bordin, Isabela Cristina Santos Freire de Paula, Jeferson Luis de Oliveira Stroparo, Samantha Schaffer Pugsley Baratto, Pablo Guilherme Caldarelli, Flares Baratto-Filho, Juliana Schaia Rocha, Marilisa Carneiro Leão Gabardo

**Affiliations:** I Universidade Positivo, School of Health Sciences, Graduate Program in Dentistry – Curitiba (PR), Brazil.; II Pontifícia Universidade Católica do Paraná, School of Life Sciences, Graduate Program in Dentistry – Curitiba (PR), Brazil.; III UniDomBosco, Department of Dentistry – Curitiba (PR), Brazil.; IV Universidade da Região de Joinville, Department of Dentistry – Joinville (SC), Brazil.

**Keywords:** Adolescent, Activities of daily living, Dental caries, Quality of life, Adolescente, Atividades cotidianas, Cárie dentária, Qualidade de vida

## Abstract

**Objective:**

To evaluate the impact of dental caries on daily activities, assessed by the Oral Impacts on Daily Performances (OIDP) and Child-OIDP in adolescents.

**Methods:**

Studies published in any language mentioning the relationship between dental caries and OIDP or Child-OIDP were included. PubMed, Scopus, Web of Science, LILACS/BBO, and grey literature were assessed to identify relevant studies published up until March 2024. The quality of the studies was assessed using the Joanna Briggs Institute tool for cross-sectional studies. For the meta-analysis and the leave-one-out sensitivity analysis, the R software was used. The subgroup analysis was conducted considering the version of the tool (OIDP or Child-OIDP) and the outcome (presence or experience of dental caries).

**Results:**

Of the 1,663 studies, 20 were included, all cross-sectional, with 17 of them conducted in schools. A total of 16 studies were considered to have high methodological quality. Individuals in the Child-OIDP and experience of dental caries subgroups showed a worse impact (PR=1.66; 95%CI 1.19–2.31; and PR=1.72; 95%CI 1.23–2.43, respectively). The heterogeneity of the studies was high (*I* ^2^=97%; T^2^=0.17; p<0.01), and we did not identify any single study as the main source for this fact in the sensitivity analysis.

**Conclusion:**

Dental caries negatively affect adolescents’ daily activities. Despite nonsignificant differences between instruments and dental caries classifications, variations in effect estimates highlight the need for further research. New studies are suggested to confirm these findings, given the high heterogeneity found (PROSPERO CRD42021247951).

## RESUMO

**Objetivo:**

Esta revisão sistemática e metanálise teve como objetivo avaliar o impacto da cárie dentária nas atividades diárias, avaliada pelo Oral Impacts on Daily Performances (OIDP) e Child-OIDP em adolescentes.

**Métodos:**

Estudos publicados em qualquer idioma mencionando a relação entre cárie dentária e OIDP ou Child-OIDP foram incluídos. PubMed, Scopus, Web of Science, Literatura Latino-Americana e do Caribe em Ciências da Saúde/Bibliografia Brasileira de Odontologia e literatura cinzenta foram acessadas para identificar estudos relevantes publicados até março de 2024. Estudos que atenderam aos critérios de inclusão foram incluídos. A qualidade dos estudos foi avaliada pela ferramenta do Joanna Briggs Institute para estudos transversais. Para metanálise e análise de sensibilidade *leave-one-out*, foi utilizado o *software* R. A análise de subgrupos foi realizada considerando o instrumento (OIDP ou Child-OIDP) e o desfecho (presença ou experiência de cárie dentária).

**Resultados:**

Dos 1.663 estudos, 20 foram incluídos, todos transversais, sendo 17 realizados em escolas. Dezesseis estudos foram considerados de qualidade metodológica alta. Indivíduos nos subgrupos Child-OIDP e experiência de cárie apresentaram pior impacto (razão de prevalência — RP=1,66; intervalo de confiança de 95% — IC95% 1,19–2,31; e RP=1,72; IC95% 1,23–2,43, respectivamente). A heterogeneidade foi alta (*I* ^2^=97%; T^2^=0,17; p<0,01), e a análise de sensibilidade não identificou nenhum estudo individual como principal fonte disso.

**Conclusão:**

A cárie dentária afeta negativamente as atividades diárias dos adolescentes. Apesar das diferenças não significativas entre os instrumentos e as classificações de cárie dentária, variações nas estimativas de efeito ressaltaram a necessidade de mais pesquisas. São recomendados novos estudos para confirmar esses achados, considerando a alta heterogeneidade encontrada (PROSPERO CRD42021247951).

## INTRODUCTION

Adolescence is a developmental stage characterized by conflicts, discoveries, and psychosocial changes that commonly lead to neglect of self-care, including oral hygiene^1^. This neglect can result in various oral problems, such as dental caries, considered quite common in this phase^2^. This morbidity is associated with poorer performance in daily activities among adolescents, including brushing teeth^3,4,5,6,7,8,9^, socializing^10,11^, sleeping^11,12^, smiling^3,8,11^, studying^11^, maintaining emotional stability^3,5,11^ and, more commonly, eating^4,5,6,7,8,9,11,12,13^.

Oral problems are increasingly recognized as integral to general health, with impacts on physical, emotional, and psychological dimensions. This has led to the concept of Oral Health-related Quality of Life (OHRQoL)^14^. Therefore, in order to measure OHRQoL, instruments called “oral health indicators” have been developed^15^. Authors of a recent bibliometric review highlighted that OHRQoL research extends beyond clinical outcomes, providing insights for public health and policies^16^.

Considering that children and adolescents differ from adults in understanding and perception, appropriate instruments are required. Chimbinha et al.^2^ identified 21 questionnaires for adolescents to assess OHRQoL. Among these, for adolescents aged 11 to 18 years, the Caregiver Perceptions Questionnaire (CPQ 11-14) and the Child Oral Impacts on Daily Performances (Child-OIDP) are recommended, with the latter being the most widely used in the literature^2^. Its multidimensional approach and applicability justify its selection in the present review.

The Child-OIDP derives from the Oral Impacts on Daily Performances (OIDP), initially designed for adults^17^, which has also been applied to adolescents^5,10,11,12,18,19,20,21,22,23^. The OIDP evaluates performance in nine aspects: eating, brushing teeth, speaking, performing light physical activities, sleeping, smiling, emotional stability, and having social contact^17^. The Child-OIDP differs from the OIDP by reordering questions and incorporating images for better understanding^24^, making it suitable for children and adolescents^3,4,6,7,8,9,11,13,25,26,27,28,29,30^. In the application, participants are asked if their teeth or mouth have caused any functional impairment (aforementioned aspects) in the last six months. If so, questions are asked about frequency, severity, perceived symptom, and physical cause of the impact. In both versions, several dental outcomes were identified by the authors as potentially impactful: toothache^3,4,7,10,19,22,25,28^, gingivitis^3,9,18,19,21,22,28,29,30^, bad breath^3,28^, and dental caries^2,3,5,6,7,8,9,10,11,12,13,18,19,20,21,22,23,26,27,28,29,30^. Furthermore, the impact of sociodemographic and socioeconomic variables^4,5,6,7,9,12,13,18,19,20,21,22,23,27,28,29,30^, habits^6,10,13,20^, and cultural factors^27^ was also observed.

Taking this into consideration, in this systematic review we aimed to assess the impact of dental caries on the performance of daily activities among adolescents aged 10 to 19 years, using the OIDP or Child-OIDP instruments.

## METHODS

### Protocol and registration

This review is registered under number CRD42021247951 in the International Prospective Register of Systematic Reviews (PROSPERO). The writing and conduction followed the recommendations of the Preferred Reporting Items for Systematic Reviews and Meta-Analyses (PRISMA)^31^ and the Joanna Briggs Institute (JBI) guidelines for evidence synthesis^32^.

### Eligibility criteria

The PECO^33^ acronym was used, according to which (P) stands for Population — adolescents; (E), Exposure — presence of dental caries; (C), Control — absence of dental caries; and (O), Outcome — the impact of oral health on the daily performance of adolescents using the OIDP or Child-OIDP instruments, to answer the question: “Does dental caries impact daily performance related to oral health in adolescents, compared to those without dental caries?”.

The inclusion criteria were: observational studies with adolescents aged 10 to 19 years, an age range defined by the World Health Organization^34^, in which the association between dental caries and OIDP or Child-OIDP was sought.

Studies that did not assess oral clinical conditions through a physical examination, those in which it was not possible to extract data from adolescents in the established age range or data related to dental caries, studies with specific populations, pilot studies, intervention studies, literature reviews, case reports, and case series were excluded.

### Search strategy and information sources

Studies were searched in the following electronic databases: MEDLINE via PubMed, Scopus, Web of Science, and the Virtual Health Library via the Latin American and Caribbean Health Sciences Literature (LILACS)/Brazilian Bibliography of Dentistry (BBO). There was no restriction regarding language or publication date. Grey literature was also searched in Google Scholar, OpenGrey, and ProQuest Dissertations and Theses. The search in Google Scholar was limited to the first one hundred articles, as the algorithm of this database prioritizes the most relevant and accessed articles (titles and abstracts) at the top of the search results^35^. In addition, in the eligible studies, a manual search was conducted in the reference lists to find any potentially unidentified studies.

The searches were conducted in January 2022, with an update in March 2024. For each database, an adaptation was made according to the specific requirements. The search strategy and the descriptors used can be found in Supplementary Material 1. The references were managed in EndNote Web (https://www.myendnoteweb.com/).

### Study selection and data extraction

Initially, duplicates were removed by two independent researchers (GMB and MCLG) by reading the titles. Then, the abstracts were read for a new selection. Subsequently, the texts were read in full when the title and abstract did not provide enough information for a decision to be made regarding eligibility. Possible disagreements between the two researchers were resolved by a third reviewer (JSR) to reach a consensus.

To extract data from the eligible studies, a form was created with the following information: author (year), country, study design, number of participants (% of men participants), age of participants, location where the study was conducted, OIDP or Child-OIDP instruments, dental caries record, and statistical analyses/adjustments. This step was also performed by two independent reviewers (GMB and ICSFP), and the data were subsequently compared.

### Methodological quality assessment

To assess the methodological quality of the studies, the JBI Critical Appraisal tools for cross-sectional studies were applied^36^. This step was independently carried out by two researchers (GMB and ICSFP; Kappa=0.79). Any disagreement was discussed with a third researcher (JSR) to reach a consensus.

Considering that there is no established method in the literature for cutoff values for classifying the level of methodological quality of the checklist for cross-sectional studies, a previous study by Amorim dos Santos et al.^37^ was used as a reference. The following classification was adopted: high, when the percentage of positive responses was up to 49%; moderate, when the values were between 50 and 69% of positive responses; and low, when there were more than 70% of positive responses.

### Meta-analysis

The meta-analysis was conducted in the R software version 4.2.2 (R Core Team 2022). Studies with sufficient data for meta-analysis were included. To this end, the Mantel-Haenszel method was used and the prevalence ratio (PR) was adopted as a measure of effect. The random effects model was used to obtain the combined effect estimate and 95% confidence intervals (95%CI). Cochran’s Q test was used to assess statistical heterogeneity between studies. The *I* ^2^ test was used to measure the proportion of variance between studies due to heterogeneity. Results of *I* ^2^≥0.75 were considered to have high heterogeneity^38^. Subgroup analysis was performed, considering the OIDP or Child-OIDP instruments, and the presence or experience of dental caries. A 5% significance level was adopted.

Sensitivity analysis was conducted in meta-analyses that included more than two studies with an *I* ^2^ greater than 40% and those identified with potential methodological differences, particularly in relation to the minimum required sample size. No studies were excluded based on methodological quality, as all included studies received high scores in the quality assessment^38^. The minimum sample size for each included study was determined based on the observed prevalence of the outcome in the study population, considering a 95%CI level and a 5% margin of error. The calculation followed the used standard methodology for cross-sectional studies, ensuring that the sample size was sufficient to provide reliable estimates^39^. Studies with insufficient sample sizes — defined as those below the calculated threshold necessary to ensure adequate statistical precision — would have been subjected to sensitivity analysis. However, all included studies met the minimum sample size requirement and, therefore, no exclusions were made based on this criterion.

A leave-one-out sensitivity analysis was conducted to evaluate the robustness of the meta-analysis results. In this approach, each study was systematically removed one at a time, and the overall effect estimate was recalculated after each exclusion. The analysis was performed separately for the overall meta-analysis and for each subgroup to examine variations in effect estimates within specific categories. The impact of each study’s removal was assessed by analyzing changes in the pooled risk ratio (RR) and 95%CI as well as fluctuations in heterogeneity statistics (*I* ^2^).

## RESULTS

**Figure 1:**
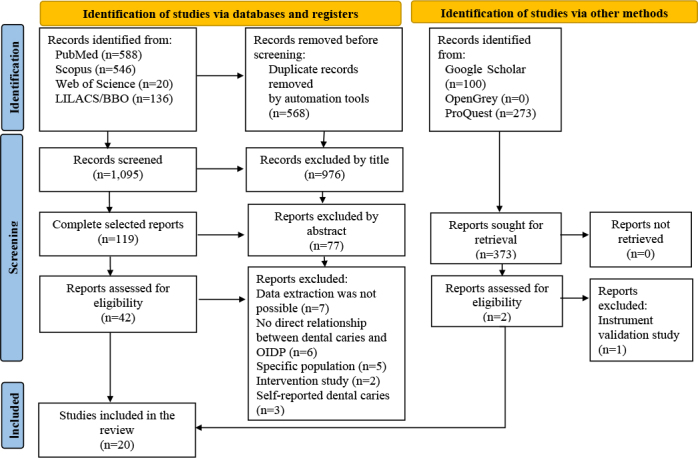
Flowchart of literature search and selection criteria.

Source: Adapted from PRISMA^31^.

A total of 1,663 studies were identified. After applying the eligibility criteria, 20 remained (Figure 1), which are summarized in Table 1.

**Table 1:** Synthesis of studies included in the review (n=20).

Age (year)	Country	Study design	Number of participants (% men)	Age (years)	Data collection site	OIDP tool	Assessed caries condition	Statistics/Adjustment
Alzahrani et al. (2019)^3^	Saudi Arabia	Cross-sectional	349 (100.0)	12–15	School	Child-OIDP	DMFT	Yes/Yes
Amilani et al. (2021)^18^	Sri Lanka	Cross-sectional	1,332 (46.8)	15–19	School	OIDP	DMFT	Yes/Yes
Anthony et al. (2023)^4^	Zambia	Cross-sectional	1,794 (46.0)	11–19	School	Child-OIDP	CAST	Yes/Yes
Bakhtiar et al. (2014)^26^	Iran	Cross-sectional	400 (46.7)	11–13	School	Child-OIDP	DMFT	Yes/No
da Cunha et al. (2017)^19^	Brazil	Cross-sectional	5,402 (43.4)	15–19	Household	OIDP	DMFT	Yes/Yes
Gajic et al. (2018)^20^	Serbia	Cross-sectional	404 (27.5)	15	School	OIDP	DMFT	Yes/Yes
Gushi et al. (2020)^21^	Brazil	Cross-sectional	5,409 (43.3)	12	Secondary data from a São Paulo oral health study	OIDP	Untreated caries	Yes/Yes
Karki et al. (2019)^27^	Nepal	Cross-sectional	1,137 (NR)	5–6, 12, 15	School	Child-OIDP	Untreated caries	Yes/Yes
Kozmhinsky et al. (2016)^22^	Brazil	Cross-sectional	1,417 (43.8)	15–19	School	OIDP	DMFT	Yes/Yes
Krisdapong et al. (2012, 2013)5,28	Thailand	Cross-sectional	12 years – 1,063 (49.6)/ 15 years – 811 (48.2)	12–15	School	Child-OIDP OIDP	DMFT	Yes/Yes
Kumar et al. (2015)^6^	India	Cross-sectional	690 (50.7)	12–15	School	Child-OIDP	DMFT	Yes/Yes
Leão et al. (2015)^10^	Brazil	Cross-sectional	180 (NR)	10–19	School	OIDP	DMFT	Yes/No
Martins et al. (2016)^12^	Brazil	Cross-sectional	389 (40.4)	10–15	School	OIDP	Untreated caries	Yes/No
Mashoto et al. (2009)^7^	Tanzania	Cross-sectional	1,745 (49.7)	10–14, 15–19	School	Child-OIDP	DMFT	Yes/No
Mohamed et al. (2019)^29^	Brazil	Cross-sectional	5,445 (48.3)	15–19	Secondary data from a São Paulo oral health study	Child-OIDP	DMFT	es/Yes
Mtaya et al. (2007)^13^	Tanzania	Cross-sectional	1,601 (39.4)	12–14	School	Child-OIDP	DMFT	Yes/Yes
Naidoo et al. (2013)^8^	South Africa	Cross-sectional	1,665 (47.0)	11–13	School	Child-OIDP	Untreated caries	Yes/No
Simangwa et al. (2020)^30^	Thailand	Cross-sectional	906 (43.9)	12–14	School	Child-OIDP	DMFT	Yes/No
Vazquez et al. (2015)^23^	Brazil	Cross-sectional	1,172 (44.0)	15–19	School	OIDP	DMFT	Yes/No
Wu et al. (2021)^9^	China	Cross-sectional	89,582 (49.5)	12–15	School	Child-OIDP	DMFT	Yes/Yes

NR: not reported; DMFT: Decayed, Missing and Filled Teeth Index; CAST: Caries Assessment Spectrum and Treatment; OIDP: Oral Impacts on Daily Performances; Child-OIDP: Child Oral Impacts on Daily Performances.

All selected studies were cross-sectional^3,4,5,6,7,8,9,10,12,13,18,19,20,21,22,23,26,27,28,29,30^. The locations and respective numbers of the studies were: Saudi Arabia^3^ (n=1), Sri Lanka^18^ (n=1), Zambia^4^ (n=1), Iran^26^ (n=1), Brazil^10,12,19,21,22,23,29^ (n=7), Serbia^20^ (n=1), Nepal^27^ (n=1), Thailand^28,30^ (n=2), India^6^ (n=1), Tanzania^7,13^ (n=2), South Africa^8^ (n=1), and China^9^ (n=1). The age of the participants ranged from five to 19 years, with a mean of 14.4 years. In eight studies, the OIDP was applied^10,12,18,19,20,21,22,23^; in 11 studies, the Child-OIDP^3,4,6,7,8,9,13,26,27,29,30^; and in one study, both^5,28^.

Data collection occurred primarily (n=17) in schools, except for three studies: one at home^19^ and two with secondary data^21,29^. The number of participants ranged from 180 individuals aged 10 to 19 years in Brazil^10^ to over 89 thousand aged 12 to 15 years in China^9^.

All studies related the OIDP and Child-OIDP to dental caries^3,6,7,9,10,12,13,18,19,20,21,22,23,26,27,28,29,30^. In 15, the decayed, missing, and filled teeth index (DMFT) was used^3,6,7,9,10,13,18,19,20,22,23,26,28,29,30^, while four studies recorded untreated caries^8,12,21,27^. Only one study employed the Caries Assessment Spectrum and Treatment (CAST)^4^. In Table 2 we summarize the extracted results, including overall mean scores with standard deviation (SD), prevalence of oral impact (%), parameters used, and their relationship with dental caries.

**Table 2:** Summary of the results extracted from the studies included in the review (n=20).

Age (year)	Mean overall score of the OIDP/ Child-OIDP (±SD)	Prevalence of oral impact (%)	OIDP/Child-OIDP Parameters	Dental caries x OIDP/ Child-OIDP
Alzahrani et al. (2019)^3^	2.15 (±1.40)	75.1	With impact/Without impact	Presence of dental caries, presence of negative impact.
Amilani et al. (2021)^18^	3.16 (±4.71)	NR	No, very little, little impact/Average impact/Severe/Very severe impact	Experience of dental caries, presence of negative impact.
Anthony et al. (2023)^4^	NR	31.5	With impact/Without impact	Experience of dental caries, presence of negative impact.
Bakhtiar et al. (2014)^26^	10.2 (±11.7)	NR	Mild impact/Moderate impact/Severe impact	Experience of dental caries, presence of negative impact.
da Cunha et al. (2017)^19^	NR	37.3	With impact/Without impact	Presence of dental caries, presence of negative impact.
Gajic et al. (2018)^20^	NR	49.5	Score	Experience of dental caries, presence of negative impact.
Gushi et al. (2020)^21^	0.93 (NR)	37.4	With impact/Without impact	Presence of dental caries, presence of negative impact.
Karki et al. (2019)^27^	2.4 (±5.0)	39.3	Without impact/Low impact/Moderate impact/High impact	Presence of dental caries, presence of negative impact.
Kozmhinsky et al. (2016)^22^	NR	66.1	Low impact/High impact	Experience of dental caries, presence of negative impact.
Krisdapong et al. (2012, 2013)5,28	NR	12 years – 81.7/ 15 years – 83.2	With impact/Without impact	Presence of dental caries, presence of negative impact.
Kumar et al. (2015)^6^	NR	36.5	With impact/Without impact	Experience of dental caries, presence of negative impact.
Leão et al. (2015)^10^	6.4 (±9.15)	NR	Score/Prevalence (%)	Experience of dental caries, presence of negative impact.
Martins et al. (2016)^12^	24.1 (±2.71)	45.6	Prevalence (%)	Presence of dental caries, presence of negative impact.
Mashoto et al. (2009)^7^	NR	36.2	With impact/Without impact	Experience of dental caries, presence of negative impact.
Mohamed et al. (2019)^29^	0.99 (NR)	39.4	Score	Presence of dental caries, presence of negative impact.
Mtaya et al. (2007)^13^	1.2 (±2.8)	28.6	With impact/Without impact	Experience of dental caries, presence of negative impact.
Naidoo et al. (2013)^8^	1.62 (NR)	36.2	With impact/Without impact	Presence of dental caries, presence of negative impact.
Simangwa et al. (2020)^30^	NR	15.8	With impact/Without impact	Experience of dental caries, presence of negative impact.
Vazquez et al. (2015)^23^	NR	NR	Score	Experience of dental caries, presence of negative impact.
Wu et al. (2021)^9^	16.1 (NR)	76.6	With impact/Without impact	Experience of dental caries, presence of negative impact.

SD: Standard deviation; NR: not reported; OIDP: Oral Impacts on Daily Performances; Child-OIDP: Child Oral Impacts on Daily Performances.

Eight studies did not report the overall mean and SD of the instrument score^5,6,7,19,20,22,23,28,30^. In four, SD was missing^8,9,21,29^, and four did not present the %^10,18,23,26^. Regarding OIDP/Child-OIDP parameters, in ten studies the outcomes were dichotomized into “with impact” and “without impact”^3,4,6,7,8,13,19,21,28,30^. Four categorized impact measurement^18,22,26,27^, five reported numerical scores^9,10,20,23,29^, and two expressed results as %^10,12^. In all studies^3,4,5,6,7,8,9,10,12,13,18,19,20,21,22,23,26,27,28,29,30^ the presence of dental caries was associated with negative impacts.

Regarding methodological quality, we present the results in Table 3. Only Naidoo et al.^8^ exhibited moderate quality, meeting five of eight parameters, while the others were classified as high quality.

**Table 3:** Assessment of the methodological quality of the evaluated studies (n=20).

Study	1. Were the criteria for inclusion in the sample clearly defined?	2. Were the study subjects and the setting described in detail?	3. Was the exposure measured in a valid and reliable way?	4. Were objective standard criteria used for measuring the condition?	5. Were confounding factors identified?	6. Were strategies to deal with confounding factors stated?	7. Were the outcomes measured in a valid and reliable way?	8. Was appropriate statistical analysis used?	Overall/ Quality
Alzahrani et al. (2019)^3^	Yes	Yes	Yes	Yes	Unclear	Unclear	Yes	Yes	75%/High
Amilani et al. (2021)^18^	Yes	Yes	Yes	Yes	Yes	Yes	Yes	Yes	100%/High
Anthony et al. (2023)^4^	Yes	Yes	Yes	Yes	Yes	Yes	Yes	Yes	100%/High
Bakhtiar et al. (2014)^26^	Yes	Yes	Yes	Yes	No	Yes	Yes	Yes	87%/High
da Cunha et al. (2017)^19^	Yes	Yes	Yes	Yes	Unclear	Unclear	Yes	Yes	75%/High
Gajic et al. (2018)^20^	Yes	Yes	Yes	Yes	Unclear	No	Yes	Yes	75%/High
Gushi et al. (2020)^21^	Yes	Yes	Yes	Yes	Unclear	Yes	Yes	Yes	87%/High
Karki et al. (2019)^27^	Yes	Yes	Yes	Yes	Yes	Yes	Yes	Yes	100%/High
Kozmhinsky et al. (2016)^22^	Yes	Yes	Yes	Yes	Unclear	Unclear	Yes	Yes	75%/High
Krisdapong et al. (2012, 2013)5,28	Yes	Yes	Yes	Yes	Unclear	Unclear	Yes	Yes	75%/High
Kumar et al. (2015)^6^	Yes	Yes	Yes	Yes	Yes	Yes	Yes	Yes	100%/High
Leão et al. (2015)^10^	Yes	Yes	Yes	Yes	Unclear	Yes	Yes	Yes	87%/High
Martins et al. (2016)^12^	No	Yes	Yes	Yes	Yes	Yes	Yes	Yes	87%/High
Mashoto et al. (2009)^7^	No	Yes	Yes	Yes	Yes	Unclear	Yes	Yes	75%/High
Mohamed et al. (2018)^29^	No	Yes	Yes	Yes	Yes	Yes	Yes	Yes	87%/High
Mtaya et al. (2007)^13^	Yes	Yes	Yes	Yes	Yes	Yes	Yes	Yes	100%/High
Naidoo et al. (2013)^8^	No	Yes	Yes	Yes	Unclear	Unclear	Yes	Yes	62%/Moderate
Simangwa et al. (2020)^30^	Yes	Yes	Yes	Yes	Yes	Yes	Yes	Yes	100%/High
Vazquez et al. (2015)^23^	Yes	Yes	Yes	Yes	Yes	Yes	Yes	Yes	100%/High
Wu et al. (2021)^9^	Yes	Yes	Yes	Yes	Unclear	Yes	Yes	Yes	87%/High

In relation to the meta-analysis, of the 20 studies, nine had sufficient data and met the inclusion criteria: six used Child-OIDP^3,5,6,9,13,28,30^, and three used OIDP^19,22,28^. Krisdapong et al.^5,28^ used both. When the presence of dental caries was considered, three studies were included^3,19,28^, and in six the experience of dental caries was the outcome^4,6,9,13,22,30^.

According to the overall effect measure (Supplementary Material 2), individuals with dental caries had significantly worse QHRQoL impact than those without it [PR=1.52 (95%CI 1.15–2.01); p<0.01]. Meta-analyses showed high heterogeneity (*I* ^2^=97%; T^2^=0.17; p<0.01). According to the leave-one-out sensitivity analysis (overall), excluding any of the studies did not substantially alter the relative risk (RR), as recalculated values remained within the original CI (Supplementary Material 3).

In the subgroup comparing the OIDP and Child-OIDP instruments, individuals assessed using the Child-OIDP showed a significant impact of dental caries on their quality of life [PR=1.66 (95%CI 1.19–2.31); Heterogeneity: *I* ^2^=96%; T^2^=0.16; p<0.01], whereas the subgroup assessed with the OIDP found no significant difference [PR=1.68 (95%CI 0.89–3.17); Heterogeneity: *I* ^2^=94%; T^2^=0.29; p<0.01], However, according to the test for subgroup differences (χ²=0.00; p=0.96), there was no difference between OIDP and Child-OIDP, suggesting that both capture the impact of dental caries on quality of life (Supplementary Material 4).

As per the leave-one-out sensitivity analysis, in the Child-OIDP subgroup, the exclusion of any study resulted in CI exceeding the original limits (blue line), demonstrating their influence on the pooled estimate. Notably, excluding Mtaya et al.^13^ and Krisdapong et al.^5,28^ extended CI beyond limits, indicating substantial impact. Conversely, in OIDP, the exclusion of Krisdapong et al.^5,28^ reduced heterogeneity, bringing CI within limits. Subsequently, removing Krisdapong et al.^5,28^ from the meta-analysis eliminated heterogeneity in OIDP subgroup (*I* ^2^=0%; T^2^=0; p=0.83), making the effect statistically significant (PR=1.21; 95%CI: 1.15–1.28). However, subgroups comparisons remained nonsignificant (χ²=1.18; p=0.28), suggesting no differences between Child-OIDP and OIDP (Supplementary Material 5).

In the subgroup analysis comparing presence versus experience of dental caries, presence yielded a pooled estimate of PR=1.19 [(95%CI 0.77–1.84); Heterogeneity: *I* ^2^=96%; T^2^=0.14; p<0.01], while experience resulted in PR=1.72 [(95%CI 1.23–2.43; Heterogeneity: *I* ^2^=97%; T^2^=0.17; p<0.01]. However, the overall test for subgroup differences was not statistically significant (χ²=1.72, p=0.19) (Supplementary Material 6).

According to the leave-one-out sensitivity analysis (Supplementary Material 7), excluding any individual study influenced the CI of the pooled estimate, as all recalculated intervals crossed the original confidence limits. In the caries experience subgroup, the exclusion of Anthony et al.^4^ and Mtaya et al.^13^ extended the CI beyond both the lower and upper confidence limits, suggesting that these studies had a significant impact on the subgroup estimate. Similarly, in the same subgroup, excluding Da Cunha et al.^19^ led to substantial effect size variation.

## DISCUSSION

In this systematic review we analyzed studies that assessed the impact of dental caries on the performance of daily activities among adolescents using the OIDP or Child-OIDP instruments. We identified an unfavorable interference in the self-perception of the participants through the use of the Child-OIDP and the experience of dental caries.

Oral problems are related to the social dimension in people’s lives, and this is especially important during adolescence, when aesthetic and physiological changes usually occur^1^, which justifies the choice of this stage of life in the present study. Furthermore, the assessment of the perception of the performance of daily activities is particularly interesting, considering that the included instruments are practical to apply and can indicate oral health needs^17,24^.

We chose the OIDP and Child-OIDP because both are applicable to adolescents^3,4,5,6,7,8,9,10,12,13,18,19,20,21,22,23,26,27,28,29,30^ and demonstrate the impact on the daily performance of relevant aspects: physical, social, or emotional^17^. They have been developed for a longer time, and the Child-OIDP is one of the most cited in research with adolescents^2^. When analyzed through these instruments, the daily activities most negatively impacted by the presence of dental caries were: brushing teeth^3,4,5,6,7,8,9^, socializing^10^, sleeping^12^, smiling^3,8^, having emotional stability^3,5^, and eating^4,5,6,7,8,9,12,13^.

The comparison between instruments suggests that Child-OIDP may be more sensitive in detecting the impact of dental caries on OHRQoL, possibly due to its adaptations for younger individuals. The observed differences between instruments, although not statistically significant, highlight the importance of considering age and cognitive factors when applying sociodental measures. The variation in age groups in each instrument may have influenced the OHRQoL. However, in the subgroup meta-analysis of the instrument, a difference between those with and without dental caries was indicated in the Child-OIDP. Among the six included studies^3,5,6,9,13,28,30^, all showed an increased risk for worse perception, with Kumar et al.^6^ reporting a value more than three times higher. The general analysis maintained this trend significantly.

Taking this into consideration, some reasons for these differences can be hypothesized. As the Child-OIDP derives from the OIDP, adaptations were necessary for individuals aged 11 years and older, including images of daily activities, response scale alterations, and simplified information^24^. One possible explanation may be an overestimated self-perception, as predicted by psychometric analyses^24^. Another reason may lie in the different age ranges used in studies, with OIDP applied to 15–19 years and Child-OIDP to 12–15 years, possibly leading to differences in responses due to cognitive development.

Another consideration is the application of both instruments at 15 years. Alzahrani et al.^3^ and Kumar et al.^6^ used OIDP, whereas Amilani et al.^18^ and Gajic et al.^20^ used Child-OIDP, contributing to heterogeneity. Moreover, an upper age limit for Child-OIDP is not mentioned in the literature, and cognitive, cultural, and socio-environmental variations in different regions may also influence the findings, despite validated and cross-culturally adapted versions being used^3,4,5,9,13,19,22,28,30^.

The distinction between presence and experience of dental caries suggests that cumulative disease burden plays a greater role in adolescents’ perception of oral health impact. Although subgroup comparison was not significant, the stronger association for experience of dental caries aligns with findings indicating that accumulated disease, rather than isolated lesions, has a greater effect on daily activities. This underscores the need to consider disease history when assessing OHRQoL.

The DMFT, reflecting accumulated disease (experience), was investigated in six studies included in the meta-analysis^4,6,9,13,22,30^. This characteristic may have contributed to differences when compared to the outcome “presence of caries” in three studies^3,5,19,28^. The meta-analysis found that only Alzahrani et al.^3^ contradicted the trend of increased risk for worse perception. Authors of a study on adolescents showed that a higher DMFT (experience) had a more negative impact on OHRQoL^40^, whereas preschoolers assessed for lesion extent (presence) showed no significant association^41^.

A total of 19 studies^3,4,5,6,7,9,10,12,13,18,19,20,21,22,23,26,27,28,29,30^ were classified as having “high” methodological quality. Naidoo et al.^8^ rated “moderate” quality for lacking clarity in sample inclusion and confounding factor handling.

During the additional sensitivity analysis, removing Krisdapong et al.^5,28^ from the meta-analysis eliminated heterogeneity in the OIDP subgroup, making the effect significant, though methodological reasons were unclear.

Nevertheless, we identified a high heterogeneity in the meta-analyses, reinforcing the need for standardized research conduct. Heterogeneity is common in systematic reviews with meta-analyses involving the use of data from observational studies. Thus, the small number of studies included in the meta-analyses did not allow for the identification of the source of heterogeneity through meta-regression, which can be considered a limitation; therefore, the findings should be considered with caution. An important reason for not including more studies in the meta-analyses is that some presented numerical results^10,12,20,23,29^, preventing data transformation. According to the subgroup analysis, only dental caries experience had a significant association with poorer quality of life, although one subgroup had only three studies, reducing statistical power.

Despite the high methodological quality of most studies, we identified substantial heterogeneity, suggesting variability in study populations, methodological designs, and measurement tools. The sensitivity analysis reinforced that multiple factors have contributed rather than a single study. The strong influence of certain studies on pooled estimates suggests that methodological choices, including sample characteristics and measurement approaches, may substantially affect the observed associations^42^.

These findings emphasize the need for standardization in future studies, particularly regarding the application of sociodental indicators and dental caries classification. Improved methodological consistency could reduce variability in and enhance comparability across studies. Additionally, the limited number of studies in some subgroups underscores the need for larger sample sizes to increase statistical power and detect differences.

As potential strengths of this review, we mention that until now there has been no similar study recorded in the literature. Its limitations include reliance on cross-sectional studies, preventing cause-effect conclusions^43^, and the exclusion of primary study authors’ input, potentially affecting the analyses. Heterogeneity in the included studies adds uncertainty to overall results. More research is needed, emphasizing standardization despite most studies being of high quality. The results can expand the discussion on oral disease impacts on OHRQoL and daily activities, guiding health policies for adolescent-focused strategies.

In conclusion, in this systematic review we showed that dental caries negatively affects adolescents’ daily activities. Although differences between instruments and caries classifications were not statistically significant, effect estimate variations warrant further research into how these factors influence self-perceived oral health impact. More studies are needed to confirm the findings given the high heterogeneity observed.
