# Supplementary material for: Impact of dental caries on the quality of life of adolescents: A systematic review and meta-analysis
Source: Rev Bras Epidemiol. 2025 May 2;28:e250018. doi: 10.1590/1980-549720250018 (PMC12054983; doi:10.1590/1980-549720250018)
Supplement: Supplementary file 1 [file 1980-5497-rbepid-28-e250018-suppl.pdf]

Supplementary material 1. Search strategy according to each database.

PubMed = 588 (04/01/2022; update 08/03/2024)		
("adolescent"[MeSH Terms] OR "adolescent"[Title/Abstract] OR "adolescences"[Title/Abstract] OR "adolescency"[Title/Abstract] OR "adolescence"[Title/Abstract] OR "adolescents"[Title/Abstract] OR "teens"[Title/Abstract] OR "teen"[Title/Abstract] OR "teenager"[Title/Abstract] OR "teenagers"[Title/Abstract] OR "teenaged"[Title/Abstract] OR "teenages"[Title/Abstract] OR "youth"[Title/Abstract] OR "youths"[Title/Abstract] OR "young people"[Title/Abstract])	("Dental caries"[MeSH Terms] OR "Dental caries"[Title/Abstract] OR "Dental decay"[Title/Abstract] OR "dental health"[Title/Abstract] OR "tooth decay"[Title/Abstract] OR "dmf"[All Fields] OR "dmft"[All Fields] OR "dmfs"[All Fields] OR "carious lesion"[Title/Abstract] OR "carious lesions"[Title/Abstract] OR "decay"[Title/Abstract] OR "caries disease"[Title/Abstract] OR "tooth caries"[Title/Abstract] OR "tooth cavity"[Title/Abstract] OR "dental cavity"[Title/Abstract] OR "cavities"[Title/Abstract] OR "caries"[Title/Abstract])	("quality of life"[MeSH Terms] OR "quality of life"[Title/Abstract] OR "OHRQoL"[Title/Abstract] OR "QoL"[Title/Abstract] OR "oral health-related quality of life"[Title/Abstract] OR "Activities of Daily Living"[MeSH Terms] OR "Activities of Daily Living"[Title/Abstract] OR "Daily Living Activities"[Title/Abstract] OR "Daily Living Activity"[Title/Abstract] OR "oral impacts on daily performance"[Title/Abstract] OR "oral impact on daily performance"[Title/Abstract] OR "OIDP"[Text Word] OR "life quality"[Title/Abstract])
#1 E #2 E #3		
Scopus = 546 (04/01/2022; update 08/03/2024)		
(TITLE-ABS-KEY (adolescen*) OR TITLE-ABS-KEY (teen*) OR TITLE-ABS-KEY (youth) OR TITLE-ABS-KEY ("young people")	TITLE-ABS-KEY ("Dental caries") OR TITLE-ABS-KEY ("Dental decay") OR TITLE-ABS-KEY ("Dental disease") OR TITLE-ABS-KEY (dmf*) OR TITLE-ABS-KEY ("carious lesion") OR TITLE-ABS-KEY (decay) OR TITLE-ABS-KEY ("caries disease") OR TITLE-ABS-KEY ("tooth caries") OR TITLE-ABS-KEY ("tooth cavity") OR TITLE-ABS-KEY ("dental cavity")	TITLE-ABS-KEY ("quality of life") OR TITLE-ABS-KEY (ohrqol) OR TITLE-ABS-KEY (qol) OR TITLE-ABS-KEY ("Activities of Daily Living") OR TITLE-ABS-KEY ("Daily Living Activity") OR TITLE-ABS-KEY ("oral impact on daily performance") OR TITLE-ABS-KEY (oidp) OR TITLE-ABS-KEY ("life quality")
#1 E #2 E #3		
Web of Science = 20 (04/01/2022; update 08/03/2024)		
TOPIC: (adolescent*) *OR* TOPIC: (teen*) *OR* TOPIC: (youth) *OR* TOPIC: ("y	TOPIC: ("dental carie*") *OR* TOPIC: ("dental decay") *OR* TOPIC: ("teeth	TOPIC: ("quality of life") OR TOPIC: (OHRQoL) OR TOPIC: (QoL) OR TOPIC:

oung people")	decay") *OR* TOPIC: (dmf) *OR* TOPIC: ("carious lesion*") *OR* TOPIC: (decay) *OR* TOPIC: ("caries disease") *OR* TOPIC: ("teeth caries") *OR* TOPIC: ("teeth cavity") *OR* TOPIC: ("dental cavit*") *OR* TOPIC: (carie*)	("oral health related quality of life") OR TOPIC: ("Activities of Daily Living") OR TOPIC: ("Daily Living Activities*") OR TOPIC: ("oral impacts on daily performance") OR TOPIC: (OIDP) OR TOPIC: ("life quality")
LILACS and BBO = 136 (04/01/2022; update 08/03/2024)		
(adolescent) OR (adolescents) OR (adolescence) OR (adolescences) OR (adolescency) OR (adolescente) OR (adolescentes) OR (teens) OR (teen) OR (teenager) OR (teenagers) OR (youth) OR (youths) OR ("young people") OR (jovem) OR (joven) OR (jóvenes)	("dental carie") OR ("dental caries") OR ("dental decay") OR (decay) OR ("tooth decay") OR (dmf) OR (dmfs) OR ("carious lesion") OR ("carious lesions") OR ("caries disease") OR ("tooth cavity") OR (cavities) OR (caries) OR (cárie) OR ("cárie dentária") OR ("caries dental") OR (CPO)	("quality of life") OR (OHRQoL) OR (QoL) OR ("oral health-related quality of life") OR ("Activities of Daily Living") OR ("Daily Living Activity") OR ("oral impacts on daily performance") OR ("oral impact on daily performance") OR (OIDP) OR ("life quality") OR ("qualidade de vida") OR ("calidad de vida")
#1 E #2 E #3		

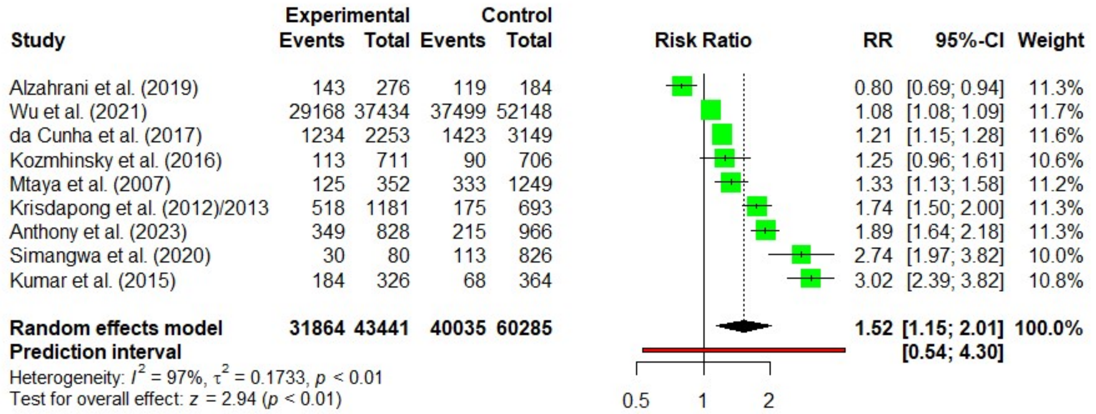

Supplementary material 2. General meta-analysis of the association between perceived impact of dental caries on daily activities of adolescents.

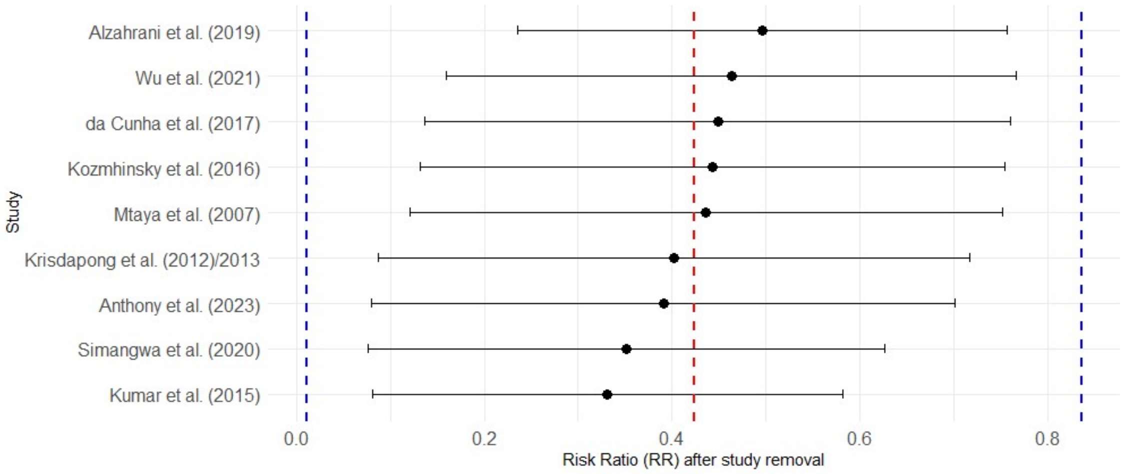

Supplementary material 3. Leave-one-out sensitivity analysis of the general meta-analysis.

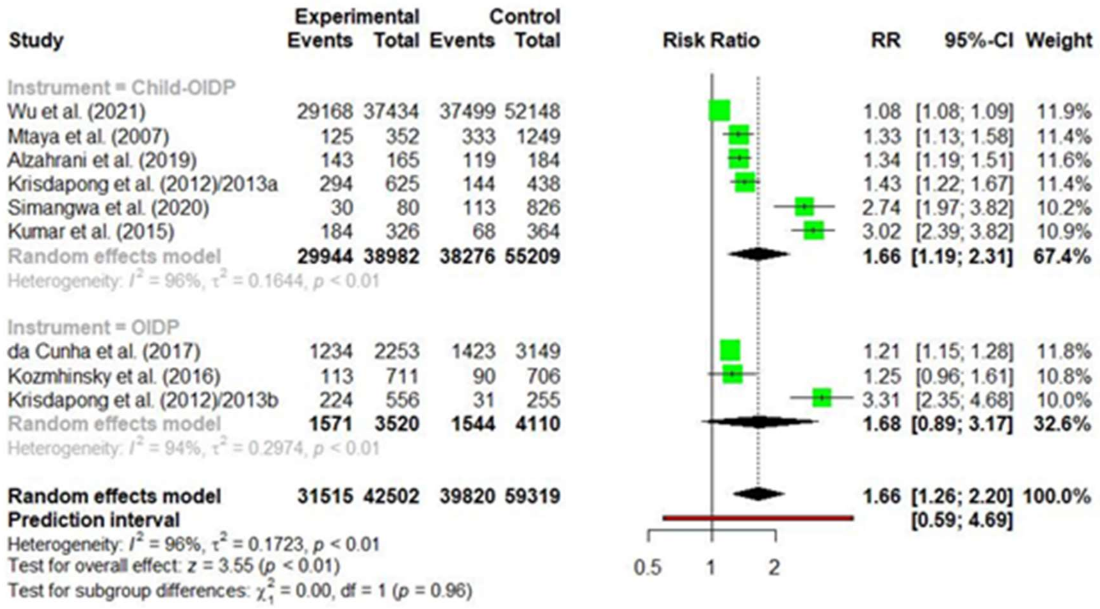

Supplementary material 4. Meta-analysis of the association between perceived impact of dental caries on daily activities of adolescents according to Child-OIDP and OIDP subgroups. Note: Experimental - with dental caries; Control - without dental caries. Different letters after Krisdapong et al.[5,28] indicate different instruments used in the studies.

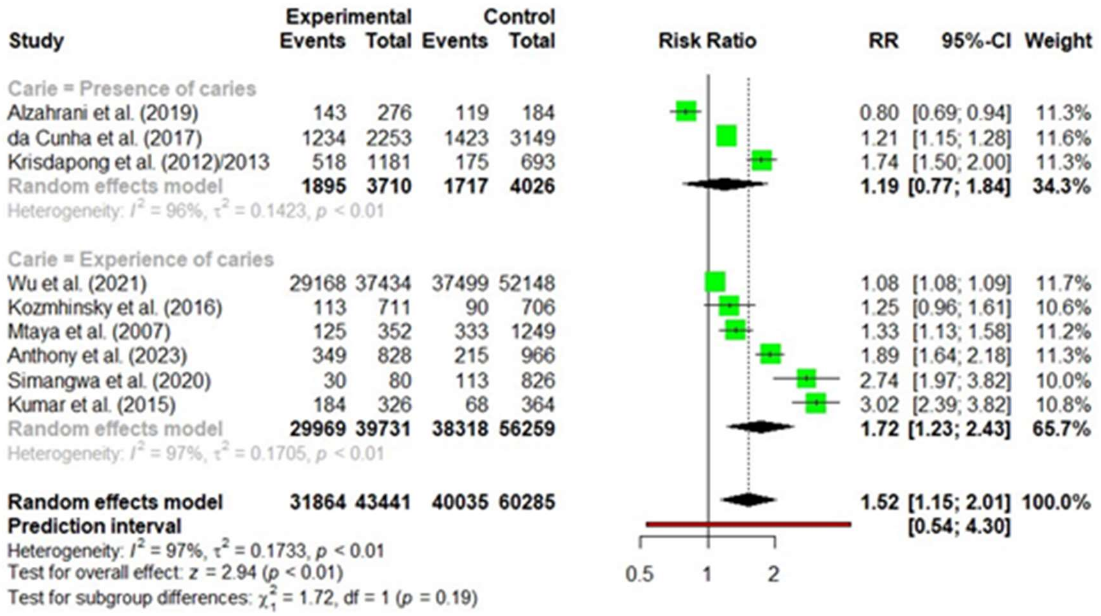

Supplementary material 5. Meta-analysis of the association between perceived impact of dental caries on daily activities of adolescents according to the presence or experience of the condition. Note: Experimental - with dental caries; Control - without dental caries. Different letters after Krisdapong et al.[5,28] indicate different instruments used in the studies.

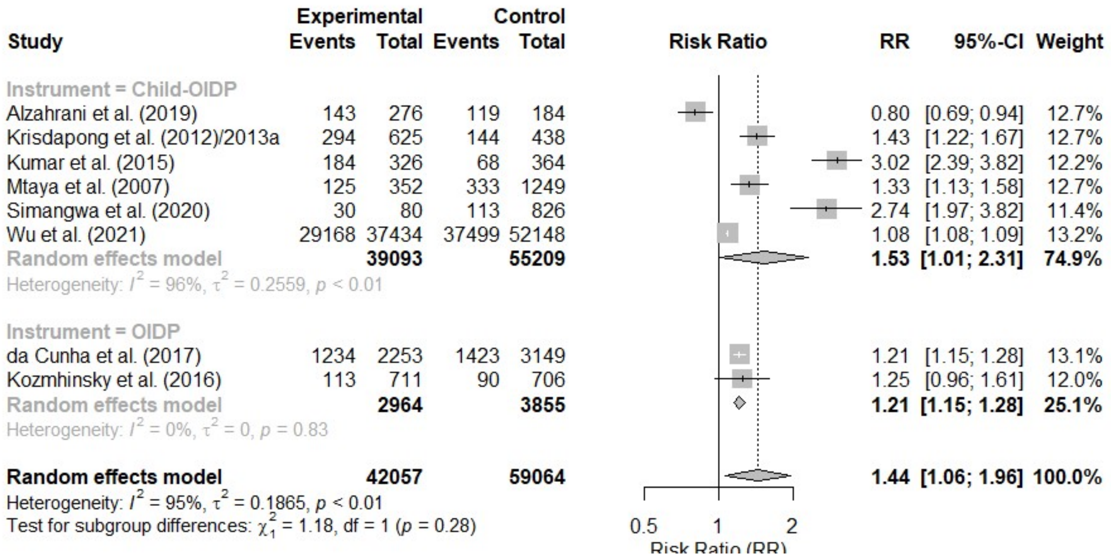

Supplementary material 6. Sensitivity analysis of the meta-analysis on the association between the perceived impact of dental caries on daily activities of adolescents, according to Child-OIDP and OIDP subgroups, after the exclusion of Krisdapong et al.[5,28]b.

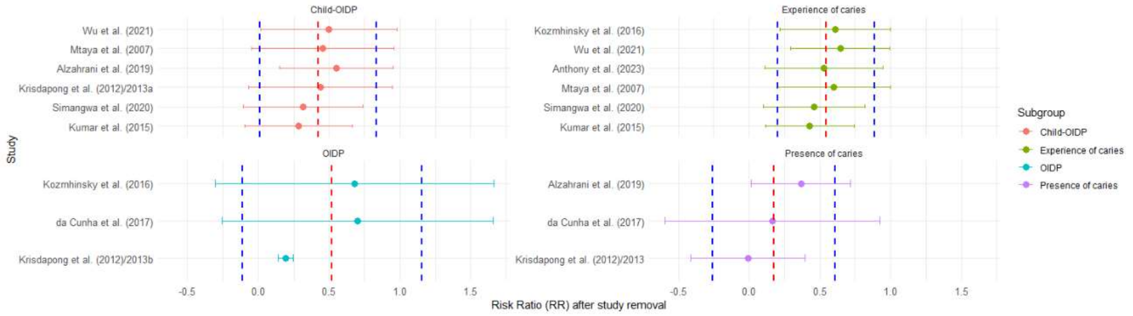

Supplementary material 7. Leave-one-out sensitivity analysis of the subgroups meta-analysis (experience of caries versus presence of caries, and OIDP versus Child-OIDP).
